# Ethanol Postpolymerization Treatment for Improving the Biocompatibility of Acrylic Reline Resins

**DOI:** 10.1155/2013/485246

**Published:** 2013-07-18

**Authors:** Cristina B. Neves, Luís P. Lopes, Helena F. Ferrão, Joana P. Miranda, Matilde F. Castro, Ana F. Bettencourt

**Affiliations:** ^1^Biomedical and Oral Sciences Research Unit (UICOB), Department of Oral Rehabilitation, Faculty of Dental Medicine, University of Lisbon, 1649-003 Lisbon, Portugal; ^2^Research Institute for Medicines and Pharmaceutical Sciences (iMed.UL), Faculty of Pharmacy, University of Lisbon, 1649-003 Lisbon, Portugal

## Abstract

*Objective*. To evaluate the effect of postpolymerization treatment based on ethanol-aqueous solutions on the residual monomer (RM) content, flexural strength, microhardness, and cytotoxicity of hard chairside reline resins (Kooliner, Ufi Gel Hard). *Methods*. After polymerization, specimens were immersed in water, 20%, 50%, or 70% ethanol solutions at 23°C or 55°C for 10 minutes. Controls were left untreated. HPLC was used for the determination of RM content. Specimens were submitted to Vickers microhardness and 3-point loading flexural strength tests. Cytotoxicity of resin eluates was determined on human fibroblasts by assessing cellular mitochondrial function and lactate dehydrogenase release. *Results*. Higher concentrations of ethanol promoted lower RM content at 55°C in both materials. The mechanical properties were maintained after 50% and 20% ethanol treatments in Kooliner and Ufi Gel Hard, respectively. Specimens submitted to those treatments showed significant reduction on cytotoxicity compared to immersion in hot water, the treatment of choice in the recent literature. *Significance*. Immersion of relined dentures in specific ethanol solutions at 55°C for 10 minutes can be considered an effective postpolymerization treatment contributing to increase materials biocompatibility. The proposed protocol is expeditious and easy to achieve with simple equipment in a dental office.

## 1. Introduction

Hard chairside reline resins are acrylic-based prosthetic biomaterials used to restore temporary, or even permanently, the fit of removable dentures when there is a change of the underlying oral tissues. They are bonded to the fitting surface of dentures reestablishing their support, stability, and retention [1]. These resins can be autopolymerized, are easy to manipulate, and gained popularity as they cure directly in the oral cavity, avoiding the additional time of expendable laboratory procedures [1]. 

During the free radical polymerization reaction, the monomer-polymer conversion is never complete [2], resulting in the presence of unpolymerized monomers in the polymer. The residual monomers (RMs) can be trapped on the polymer matrix, affecting the mechanical properties of the material [2] or can be diffused into the surrounding medium, causing undesirable biological reactions [3], including chemical irritation, hypersensibility, mucosal inflammation, vesiculation and ulceration, burning sensation, and systemic allergic reactions [3–5]. 

The curing process of these kinds of autopolymerizing resins is achieved in direct contact with the oral mucosa, leading to high levels of RM content once the material had set *in vivo* [6, 7]. As the RM content is often associated with the quantity of RM leached to the surrounding media [3–6, 8, 9], the possible high RM elution, initiated during the curing of the material, led to an increasing concern of the scientific community about the toxicological consequences related to the use of these resins [10]. The search for effective methods of postpolymerization treatments that decrease the RM content became relevant [7, 11–13].

In recent years, different postpolymerization treatments have been proposed to reduce the oral exposure to the RM and the degradation products of reline acrylic resins, including immersion in hot water [5, 7, 11–14] and microwave irradiation [7, 11, 12, 14]. The proposed treatments reduce RM content [7, 12, 13] and decrease the leachability of residual compounds to the media [5, 13] with the purpose of minimizing resins cytoxicity and improving their biocompatibility [5, 7, 10, 11, 13, 15]. 

To date, in the mentioned studies, water has been used as the postpolymerization immersion medium. Generally, apart from water, ethanol aqueous solutions have been used in order to increase and accelerate compounds solubility, indicating the importance of this solvent in leaching processes [3, 16–19]. Bettencourt et al. [20] showed that ethanol increases the RM leaching from the polymer matrix of acrylic bone cements used in joint arthroplasty. Other studies found that immersion in pure ethanol (99.5%) promoted a reduction of the residual compounds content on acrylic polymers used in dentistry as denture base resins [18] and temporary restorative resins [19]. Ethanol molecules penetrate the material matrix and expand the space between polymer chains into which insoluble substances may diffuse [18]. Ethanol also accelerates water sorption to the polymer matrix, promoting the RM diffusion from the polymer [21]. These facts led the authors to consider ethanol aqueous solutions as a possible practical vehicle for removing RM from hard chairside reline resins and therefore improve their biocompatibility. 

Since water immersion postpolymerization treatments are dependent on temperature [7, 11–13], our experiments also enclosed the possible benefits of the interaction between ethanol aqueous solutions and temperature. In fact, temperature is known to promote an additional polymerization of the resins decreasing RM content [7, 12, 14].

The hypothesis of this work was that postpolymerization treatment based on immersion in ethanol solutions would improve the biocompatibility of two different hard chairside reline resins. Biocompatibility can be defined in terms of the ability of a material to perform a specific function with an appropriate host response [22]. Specifically, an increase of biocompatibility means less toxic effects, while the material keeps its mechanical properties. Increase of biocompatibility as a consequence of a novel proposed postpolymerization treatment was assessed considering three key aspects of the reline resins: RM content, mechanical properties, and cytotoxicity.

## 2. Materials and Methods

The materials evaluated in this study are presented in Table 1 and represent two known autopolymerizing hard chairside reline resins. Both are composed of poly(ethylmethacrylate) polymer but have distinct monomers: isobutylmethacrylate (IBMA) and 1,6-hexanedioldimethacrylate (1,6-HDMA) in Kooliner and Ufi Gel Hard, respectively, [23].

### 2.1. Hard Chairside Reline Resins Specimens Preparation

Specimens of each material were prepared from stainless steel molds as ISO 20795-1 recommends [24]. The materials were prepared according to the manufacturers' recommendations (Table 1), and the mixture was placed into the metal mold (disk or rectangular shaped). The mold and the materials dough were maintained under compression, between two glass plates, at 37 ± 2°C during the recommended polymerization time (Table 1), in order to simulate the intraoral polymerization of the material. The obtained specimens were then used for the determination of the RM content (Section 2.2), mechanical tests (Section 2.3), and cytotoxicity assays (Section 2.4) and were treated accordingly. 

### 2.2. Determination of RM Content

Specimens of each material with a diameter of 50.0 ± 0.1 mm and thickness of 3.00 ± 0.01 mm were randomly divided into ten groups of 6 specimens each (*n* = 60). Each specimen was exposed during 10 minutes to the postpolymerization treatment. Two groups were exposed to dry conditions: one at 23 ± 2°C (control group) and the other at 55 ± 2°C. All the other specimens were exposed in closed plastic flasks to water or one of the three ethanol/water solutions of 20, 50, and 70% (*V*/*V*) at 23 ± 2°C or 55 ± 2°C [20]. Postpolymerization treatments at 23 ± 2°C simulate room temperature and show the individual effect of the solvent on reducing the RM content of the resins. Experiments at 55 ± 2°C permitted to explore the synergetic effect of temperature and solvent on reducing the RM content. 

After the postpolymerization treatments, all specimens were milled into small pieces in order to prepare three samples of each specimen. To each sample, of approximately 300 mg, 5 mL of acetone was added (extraction solvent) [24]. The sample solutions were magnetically stirred for 72 hours. To precipitate the dissolved polymer, 8 mL of methanol was added to 2 mL of each of the previously prepared samples. The slurry was then centrifuged at 10.000 rpm for 10 minutes, and 20 *μ*L of aliquots of the supernatant from each solution (triplicates) was used for quantification of the monomers by HPLC (Shimadzu system LC-6A; RP-18-Lichrospher-Merck column; mobile phase of acetonitrile/water (60 : 40); flow rate of 1 mL/min; UV detection at 230 nm).

Total quantity of RM (*μ*g) in 1 g of each sample was calculated according to the following equation: *m*_RM_ = [*c*_RM_ (*μ*g/mL) × 100 mL × (5 mL/2 mL) × (1000 mg/*m*_sample_)], where *c*_RM_ is the concentration of the RM in the solutions analysed by HPLC and *m*_sample_ is the mass of the sample in micrograms [24].

### 2.3. Mechanical Tests

The mechanical tests were carried out only on the groups submitted to 55 ± 2°C, since they showed significant reduction of the RM content.

Specimens of each material (64 × 10 × 3.3 mm) obtained as described above (see Section 2.1) were randomly divided into five groups of eight specimens each (*n* = 40). One group was left untreated (control group) while the other groups were submitted to the correspondent treatment (water, 20%, 50%, or 70% ethanol solutions) at a temperature of 55 ± 2°C for 10 minutes. Before testing, all specimens were bench-cooled to room temperature and stored in water at 37 ± 2°C, for 48 ± 2 hours, as recommended by ISO 20795-1 [24].

Each specimen was tested for both microhardness and flexural strength values. Microhardness of the specimens was tested prior to flexural strength, since the load applied for fracture could create superficial tension forces that can be propagated and interfere with the microscopic measures of superficial microhardness.

#### 2.3.1. Vickers Microhardness Test

The microhardness of the specimens was obtained using a Vickers diamond indenter attached to a microhardness indenter machine (Duramin, Struers DK 2750 Ballerup, Denmark) using a 25 gf (245 mN) load for 30 seconds, as described elsewhere [25]. The lengths of the diagonals were taken immediately after each indentation, with a minimal period of time (as short as 10 seconds) between making and reading the indentations, therefore, assuming that the viscoelastic recovery of the material was minimal. The equipment automatically converted these measurements to Vickers microhardness numbers (VHNs) expressed in kg/mm^2^. Twelve indentations were made on each specimen. 

#### 2.3.2. Flexural Strength Test

After microhardness testing, all specimens were submitted to flexural strength test in a servo-hydraulic universal machine (Instron Model 4502) using 3-point loading. A crosshead speed of 5 mm per minute was used and the distance between the supports was 50 mm, as described elsewhere [25]. The average of individual measures (width and thickness) of each specimen was introduced in the software just before testing. 

Load was applied until failure and the fracture load was recorded in Newton (N). The flexural strength was expressed in megapascal (MPa) and calculated using the following formula: FS = 3*WL*/2*bd* ^2^, where FS is the flexural strength, *W* is the maximum load before fracture (N), *L* is the distance between the supports (50 mm), *b* is the width of the specimen (mm), and *d* is the thickness of the specimen (mm). 

### 2.4. Cytotoxicity Assays

Evaluation of cytotoxicity was assessed only on the groups of the specimens submitted to water and ethanol solutions (55 ± 2°C) that were considered effective postpolymerization treatments on reducing RM content while keeping the mechanical properties of the materials. These tests were carried out with the extracts [26]. Disk-shaped specimens (diameter of 50.0 ± 0.1 mm and thickness of 2.00 ± 0.01 mm) of each material (*n* = 9) prepared as previously described (see Section 2.1), under aseptic conditions, were divided into three groups: (1) without treatment (W/T), (2) heat-treated in a water bath at 55 ± 2°C for 10 min, and (3) heat-treated in a specific ethanol solution (50% for material K and 20% for material U, at 55 ± 2°C for 10 min).

Eluates of each material were prepared by placing each disk into a sterile glass vial with 25 mL of Dulbecco's Modified Eagle's medium (Sigma-Aldrich), supplemented with penicillin-streptomycin and fetal bovine serum (Sigma-Aldrich). Disks were incubated at 37°C for 24 h. A medium without disks was also incubated as above to serve as the control medium [26].

The *in vitro* cytotoxicity of the eluates was investigated in human Adult Dermal Fibroblast Cells (Zen-Bio Inc., Chapel Hill, USA). Briefly, cells were seeded on a 96-well plate at a cell density of 3.2 × 10^3^ cells/well, 24 h previously to the cytotoxicity tests and incubated at 37°C and 5% CO_2_ in a humidified atmosphere. Cells were then incubated with the eluates or controls. After 24 h, the cell viability was analyzed by the 3-[4,5- dimethylthiazol-2-yl]-2,5-diphenyl tetrazolium bromide (MTT) reduction assay (see Section 2.4.1), and the supernatants were removed and stored at 4°C for the Lactate dehydrogenase (LDH) assay (see Section 2.4.2).

#### 2.4.1. MTT Assay

Cell viability was determined by the ability of the cells to metabolically reduce the tetrazolium salt (MTT) to a purple formazan dye [26]. Briefly, 200 *μ*L of the MTT dye solution (0.5 mg/mL) was added to each well. After 2.5 h, formazan crystals were solubilized and extracted with dimethylsulfoxide (DMSO). After 15 min at room temperature, the absorbance was measured at 595 nm and results were expressed as % of the control (culture medium).

#### 2.4.2. LDH Assay

Cytotoxicity based on quantifying the release of LDH from membrane-damaged cells was determined using the assay kit TOX7 provided by Sigma-Aldrich, which measures the conversion of a tetrazolium salt into a red formazon product (absorbance was recorded at 490 nm). The percentage release of LDH from the treated cells was calculated by comparing it to the maximum release of LDH achieved by incubating the cells with a DMSO solution (20%).

### 2.5. Statistical Analysis

Statistical analysis for RM content consisted of a two-way analysis of variance (ANOVA) followed by Tukey multiple means comparisons (at a *P* < 0.05 level).

Data of microhardness, flexural strength tests, and cytotoxicity assays were analyzed by Kruskall-Wallis test and individual differences were investigated by Tukey test (both at a *P* < 0.05 level).

## 3. Results

### 3.1. RM Content

The RM content of K and U showed higher quantities of residual IBMA compared with residual 1,6-HDMA (Table 2). In both materials, the two-way ANOVA analysis of RM content data found significant interaction between temperature and concentration of ethanol (*P* < 0.001). Specimens submitted to the higher temperature presented significantly lower values of RM content (*P* < 0.001) (Table 2), except for groups submitted to dry conditions, where *P* = 0.535 and *P* = 0.747 for K and U, respectively. 

For both materials, higher concentrations of ethanol led to lower levels of RM content when submitted to 55 ± 2°C (*P* < 0.001), except for the 20% ethanol group of material K that had the same results as the water group (Table 2).

### 3.2. Microhardness

**Figure 1:**
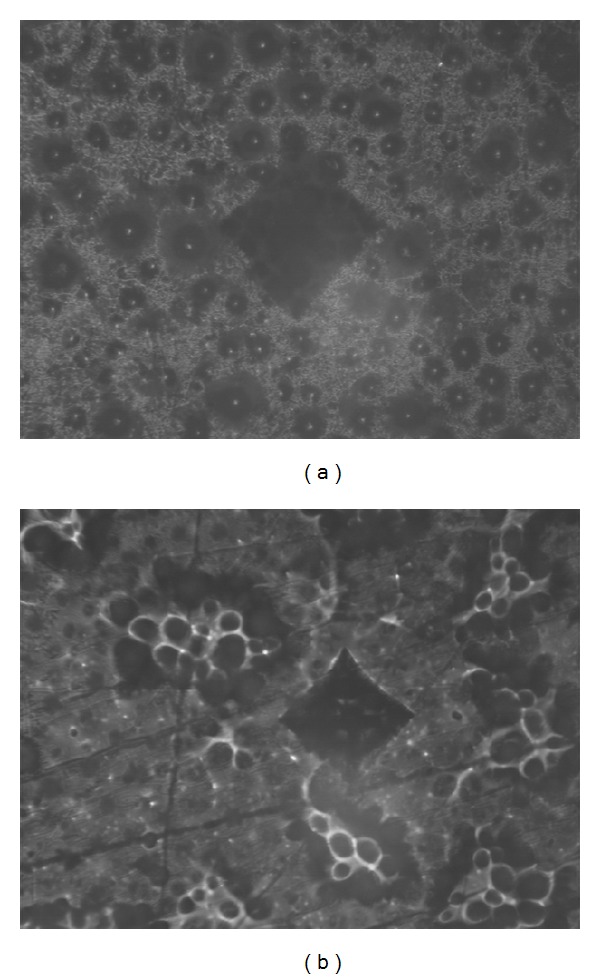
Microscopic images of Vickers indentations produced on specimens from the ethanol 70% groups submitted to 55 ± 2°C; (a) = K specimen, (b) = U specimen.

All specimens of the 70% ethanol group from both materials presented an irregular surface that prevented the determination of Vickers microhardness (Figure 1).

**Figure 2:**
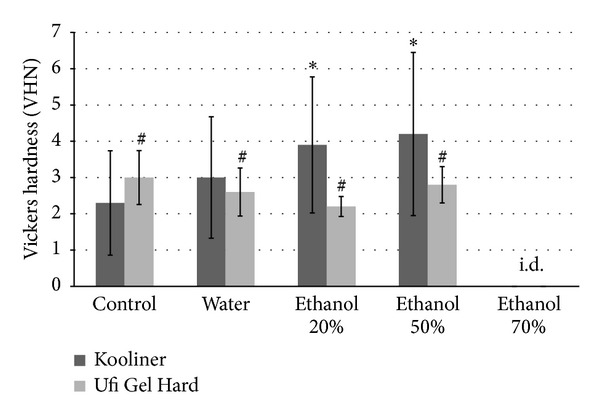
Mean and SD of Vickers microhardness (VHN) of K and U experimental groups (*n* = 8). i.d.= impossible determination. Identical characters, (∗) for K and (#) for U denote no significant differences among groups (*P* > 0.05).

Considering K specimens (Figure 2), significant differences in microhardness were found between groups (*P* < 0.001). All of the experimental groups presented higher values of microhardness than the control group (*P* < 0.05). The 20% and 50% ethanol groups showed significant higher values than water group (*P* < 0.001). For U specimens (Figure 2), no differences in microhardness were found significant between groups (*P* > 0.05). 

### 3.3. Flexural Strength

**Figure 3:**
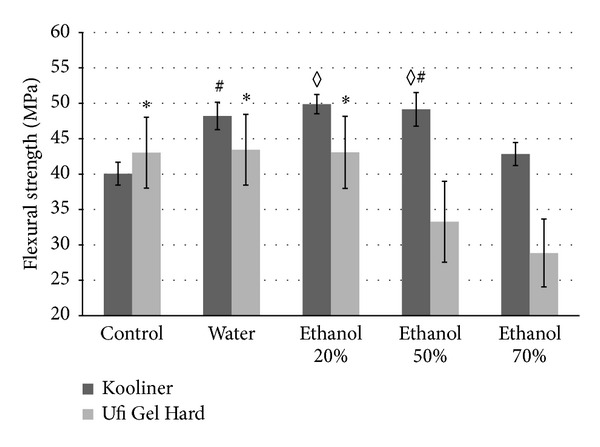
Mean and SD of flexural strength (MPa) of K and U experimental groups (*n* = 8). Identical characters, (#) and (*◊*) for K and (∗) for U denote no significant differences among groups (*P* > 0.05).

For K specimens (Figure 3), all experimental groups presented significant higher values of flexural strength than the control group (*P* < 0.001). Comparing to water, the 70% ethanol group showed a significant reduction on flexural strength (*P* < 0.001); the 50% ethanol group had no differences (*P* = 0.484), and the 20% ethanol group had higher values (*P* = 0.019). 

For U specimens (Figure 3), no differences were found in water and the 20% ethanol groups when compared to the control group (*P* > 0.05). Both the 50% and the 70% ethanol groups had significant lower values of flexural strength than the other groups (*P* < 0.001).

### 3.4. Cytotoxicity Assays

**Figure 4:**
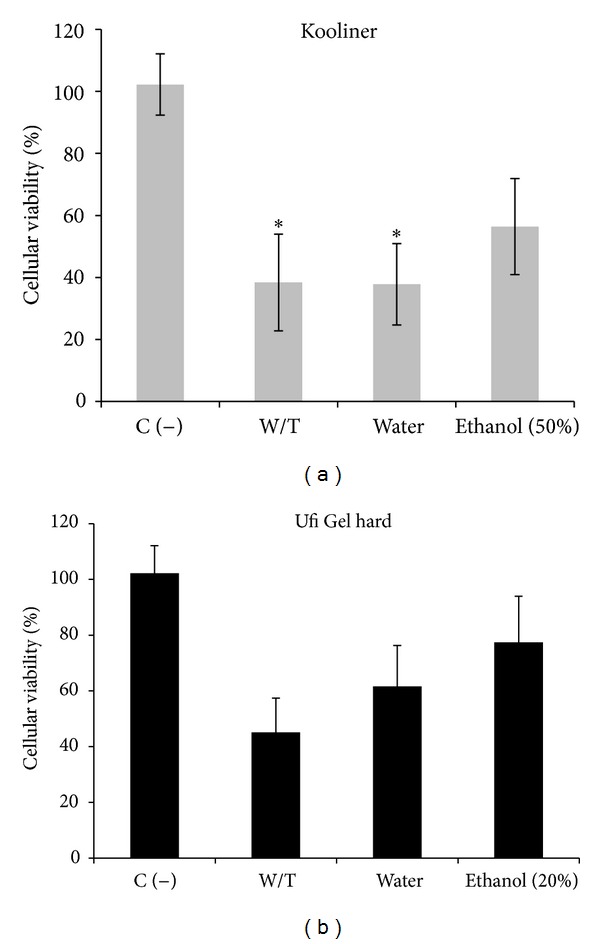
Effect of postpolymerization treatment on cytotoxicity assessed by the MTT test. Values represent mean and SD of at least three experiments. W/T = without-treatment group. C(−) = Incubation of cells with culture medium. Identical characters (∗) denote no significant differences among groups (*P* > 0.05).

**Figure 5:**
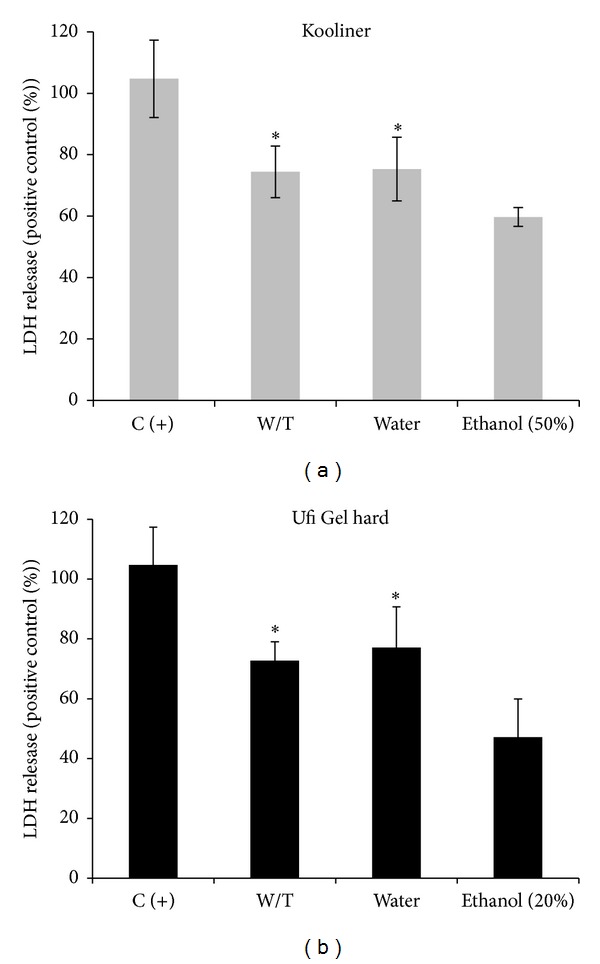
Effect of postpolymerization treatment on cytotoxicity assessed by the LDH release assay. Values represent mean and SD of at least three experiments. W/T = without-treatment group. C(+) = incubation of cells with 20% DMSO. Identical characters (∗) denote no significant differences among groups (*P* > 0.05).

For both materials, eluates obtained from ethanol-treated groups resulted in higher cell viability compared to without-treatment (dry conditions) and water-treated groups (Figures 4 and 5). 

Cell viability determined by the ability of cells to metabolically reduce MTT to a formazan dye showed that postpolymerization treatment of the K resin with ethanol increased the cell viability from ~38% to ~56% (compared with water treatment). In what refers to U resin, ethanol treatment increased the viability from ~62% to ~77% (Figure 4). 

In the case of LDH release, ethanol-treated groups showed a significant decrease compared to positive control, 59.7 ± 3.1% and 63.7 ± 14.3% for materials K and U, respectively, (Figure 5). Moreover, a significant increase in % LDH release was observed for the without-treatment and water-treated groups when compared to ethanol groups. 

## 4. Discussion

 In the present work, the effect of ethanol solutions as postpolymerization treatment was evaluated in order to improve the biocompatibility of two distinct hard chairside reline resins (K and U). For this purpose, different parameters were tested and monitored, namely, the RM content, the microhardness, and flexural strength of the material, as well as the cytotoxicity of the corresponding eluates.

The RM content of both K and U was quantified by HPLC showing higher quantities of residual IBMA compared with residual 1,6-HDMA, respectively, in all conditions evaluated. These results are in accordance with previous studies [7, 13] that described K to be the material with the highest level of RM, regardless of the experimental conditions. These findings can be explained by differences found in the resin matrix composition. The IBMA monomer is a monofunctional methacrylate monomer in opposition to the dimethacrylate monomer 1,6-HDMA. Bifunctional monomers might improve the polymerization process by providing more reactive groups and the extent of the polymerization reaction [2]. Also, 1,6-HDMA is a cross-linking agent that shows a large distance between the two methacrylate groups, which could increase the reactivity of the second methacrylate group resulting in a more complete polymerization and lower levels of RM [23]. 

Moreover, an important finding of our study was the reduction of the RM content of both specimens, at 55°C, due to ethanol solutions treatment when compared with water. Indeed, it has been previously suggested that the chemistry of different solvents is a key element in postpolymerization treatments [17], as it influences monomer solubility in the extraction media. The correlation between the chemistry of a solvent and monomers solubility may be assessed through the Hildebrand solubility parameter (*δ*) which provides a numerical estimate of the degree of interaction between compounds [27]. Compounds with similar values of *δ* are likely to be miscible. The *δ* of the monomers is ~16.0 MPa^1/2^, which is closer to the ethanol (*δ* = 26.0 MPa^1/2^) than to the water (*δ* = 47.9 MPa^1/2^). This fact may explain why a higher proportion of ethanol in the solution leads to a more significant reduction of RM content, since ethanol solutions (20, 50, and 70%) progressively approximate 100% ethanol. Also, Bettencourt et al. [20] found that the amount of RM released from acrylic polymers was linearly related to ethanol concentration. 

 The synergic effect of high temperature and ethanol solutions on reducing the RM content was evaluated as well. High temperature (~55°C) has already been considered a crucial element in the postpolymerization treatment of acrylic resins, since it seems to be responsible for a further consumption of RM during polymerization [7, 12] and allows for an earlier saturation of the solution sorption, increasing RM diffusion to the medium [5, 12]. In fact, our results indicate that a postpolymerization treatment based on a combined approach of ethanol solutions and high temperature (55°C) reduces the RM content of reline resins specimens. To the best of our knowledge, it is the first time that such a fact has been shown. In spite of not being an isolate causing factor, the RMs present in the acrylic resins and leached into the medium are considered potential toxic agents that can promote adverse reactions on tissues of the oral cavity [3]. Thus, the decrease on RM content is highly relevant in what concerns possible harmful effects of the resins on the oral mucosa, as it is associated with a decrease in the quantity of RM leached to the oral environment [3–6, 8, 9].

Another important consideration is the impact of these novel proposed postpolymerization treatments on the mechanical properties of the resins. Replacement of RM molecules with solvent molecules has been associated with a plasticizing effect observed in postpolymerization treatments [28, 29], whereas temperature compensates this plasticizing effect by increasing the rigidity of the material [12–14, 16, 19–21, 30]. The equilibrium between these two factors dictates the effect of a postpolymerization treatment on the mechanical properties of the materials. In this study, this effect was assessed by microhardness and flexural strength measurements. A decrease on these measures reveals a negative effect of the postpolymerization treatment on the mechanical properties of resins.

In the present study, immersion in water at 55°C of K specimens produced a significant increase of their microhardness and flexural strength compared with the controls. This can be explained by a stronger effect of the temperature compared with the water plasticizing effect. Nevertheless, U specimens did not show any differences on microhardness and flexural strength after hot water bath (at 55°C), possibly because of differences in the polymeric structure between the two resins. Material U undergoes rapid polymerization reaction and solidifies quickly. It is likely that air voids are entrapped during mixing of the powder and liquid components, which result in a porous structure [7, 13, 14]. This porous structure can promote the migration of water molecules, weakening the polymer net, and balancing the effect of temperature [19].

When determining the most effective postpolymerization treatment, the one that reduces the RM content more effectively should be chosen. However, in this choice, professionals must also consider if the mechanical properties of the resin are not negatively affected. Since a water bath at 55°C has already proven to be an effective postpolymerization treatment to reduce the RM content of materials K and U [10], the ethanol treatment presently proposed should be even more effective than water on the reduction of the RM content. Specimens of both materials from the 50% and the 70% ethanol groups showed more reduction in RM monomer than water, 70% ethanol group being the more effective solution. Nevertheless, this treatment produced internal weaknesses of the materials. Immersion on 70% ethanol solution showed a macroscopic image of holes on the resins suggesting that ethanol can enhance the size of the inner porous, promoting significant changes on resins network structure. This was further confirmed by a reduction of the flexural strength of the specimens, when compared to water treatment. Also, highly porous surface precluded the microscopic examination of the Vickers indentation. Thus, since this treatment produced internal weakness of the materials, it was considered unvaluable as a postpolymerization treatment. So, the 50% ethanol solution should be the treatment of choice for K resin, promoting a more effective reduction in RM content than water, while maintaining the mechanical properties of the resin. For the material U, the 50% ethanol solution produced a reduction on the flexural strength of the resin. In this matter, the 20% ethanol solution at 55°C was found to reduce the RM content more effectively than the water, without any degradation of the specimens, thus, being the treatment of choice for the U resin. As previously stated, divergent results between K and U resins may be explained due to differences in the structure of polymer network.

The clinical success of acrylic resins depends not only on the chemical and mechanical properties of the materials but also on their biological safety. As such, the postpolymerization treatment that proved to promote a higher reduction in RM content while maintaining the mechanical properties (i.e., 50% or 20% ethanol solution at 55°C, for K and U, resp.) was further evaluated in what concerns cytotoxicity.

In this study, *in vitro* assays were used to determine the effect of resin eluates on human fibroblasts viability as a measure of material's cytotoxicity. Human fibroblasts are a cell model used in cytotoxicity assays due to its reproducible and high growth activity [4, 31]. In addition, since acrylic resins are in intimate contact with a large area of dry and fragile oral mucosa as it happens with xerostomia, where ulceration of epithelium frequently occurs after denture placement, the use of human fibroblasts may have an enhanced relevance [32]. 

Two different endpoints commonly used as a measure of dental materials cytotoxicity, mitochondrial enzyme activity (MTT assay), and plasmatic membrane damage (LDH assay) were used to assess the *in vitro* toxicity of resins to fibroblast cells [3, 33, 34]. The combination of different methods with specific targets within the structure of the cell is highly recommended since it provides a more reliable final evaluation of cytotoxicity [11]. 

In the present study, the results from both cytotoxicity assays were consistent and showed that the use of ethanol aqueous solutions at 55°C as postpolymerization treatment significantly decreased the cytotoxicity of both materials. In contrast, immersion in water at 55°C had no significant effect on materials cytotoxicity. Similar results were reported by others who found that postpolymerization heat-water treatments did not markedly influence *in vitro* cytotoxicity regardless of reducing RM content and decreasing the leachability of residual compounds [5, 7, 11–13, 35]. The high decrease of RM content due to ethanol treatment compared to water may partially explain the greatest impact of the novel postpolymerization treatment on materials toxicity. Moreover, it is known that *in vitro *cytotoxicity might not only be the result of the leaching RM but also other components such as additives, byproducts, impurities, and decomposed products [11, 18] with enhanced solubility in ethanol-water solutions. 

 At this point, we may conclude that the hypothesis of our study was found to be partially accepted, since postpolymerization treatments based on ethanol solutions did improve the biocompatibility of the acrylic resins in the groups submitted to a combination approach of ethanol-water solutions and temperature.

Autopolymerized hard reline resins are commonly used for direct relining of dentures. Advantages of time, cost, and logistics of these acrylic resins compared to the laboratory-processed reline systems became very relevant in the oral rehabilitation of a growing geriatric and frail population. However, the biocompatibility of these materials is still a problem [36–38]. Different monomer reduction techniques are being under evaluation with the aim of improving materials biological behaviour. So far, a combination of temperature and ethanol-water solutions has never been tried. Within the limitations of our experimental protocol due to the fact that this study was conducted *in vitro *with one single-cell type (fibroblasts), the results showed that the novel proposed postpolymerization treatment might have a significant impact on reducing RM content and toxicity of relining materials, indicating that it could be used to improve their biocompatibility. A further advantage is the fact that the proposed postpolymerization treatment is expeditious and easy to achieve with simple equipment in a dental office.

## 5. Conclusion

Under our experimental conditions, a postpolymerization treatment based on a combination approach of ethanol-water solutions and temperature enabled the reduction of the monomer content and the cytotoxicity of acrylic reline resins, while keeping their mechanical properties. Specifically, for Kooliner, the immersion on 50% ethanol solution at 55°C during 10 min showed to be the best condition. In Ufi Gel Hard, the most effective postpolymerization treatment was the 20% ethanol solution at 55°C during 10 minutes.

## Figures and Tables

**Table 1:** Materials under evaluation in the study.

Product	Manufacturer	Batch number	P/L ratio (g/mL)	Composition	Curing cycle
Kooliner (K)	GC America Inc., Alsip, IL, USA	0701222(P); 0704052(P); 0708151(P); 0610041(L).	1.4/1	P: PEMA L: IBMA	10 minutes

Ufi Gel Hard (U)	Voco GmbH, Cuxhaven, Germany	0905422(P); 771715(P); 0905421(L); 760494 (L).	1.77/1	P: PEMA L: 1,6-HDMA	7 minutes

P: powder; L: liquid; PEMA: poly(ethylmethacrylate); IBMA: isobutylmethacrylate; 1,6-HDMA: 1,6-hexanedioldimethacrylate.

**Table 2:** Mean (×10^4^ *µ*g/g) and SD of RM content of experimental groups from material Kooliner (*n* = 6) and Ufi Gel Hard (*n* = 6).

Material	Temp.	Dry conditions	Type of solution			
			Water	Ethanol 20%	Ethanol 50%	Ethanol 70%
Kooliner	23 ± 2°C	2.77 (0.29)^aA^	2.51 (0.16)^aA^	2.56 (0.25)^aA^	2.57 (0.31)^aA^	2.46 (0.41)^aA^
	55 ± 2°C	2.67 (0.22)^aA^	2.04 (0.21)^bB^	1.88 (0.15)^bB^	1.59 (0.14)^cB^	0.80 (0.16)^dB^

Ufi Gel Hard	23 ± 2°C	1.90 (0.15)^aA^	1.62 (0.26)^bA^	1.58 (0.17)^bA^	1.52 (0.23)^bA^	1.45 (0.11)^bA^
	55 ± 2°C	1.85 (0.32)^aA^	0.95 (0.05)^bB^	0.86 (0.42)^cB^	0.72 (0.13)^dB^	0.39 (0.05)^eB^

Horizontally identical superscripted small letters denote no significant differences among groups (*P* > 0.05).

Vertically identical superscripted capital letters denote no significant differences among groups (*P* > 0.05).
